# Commentary: Localization of contractile wave nucleation sites as an emerging phenomenon of stochastic myogenic gut motility

**DOI:** 10.3389/fcell.2022.979646

**Published:** 2022-08-29

**Authors:** Nicolas R. Chevalier

**Affiliations:** Laboratoire Matière et Systèmes Complexes, Université Paris Cité, CNRS UMR 7057, Paris, France

**Keywords:** chicken, gut, peristalsis, myogenic, refractory period

In their recent article, Shikaya et al. (2022) reported on the existence of preferential sites of nucleation of contractile waves along the chicken embryonic day 12 midgut, termed sites of “origin of peristaltic waves” (OPW). Histological analysis by the authors did not reveal any peculiarities of these sites compared to other locations along the gut. Applying tetrodotoxin or ablating neural crest cells did not suppress the OPWs, but led to a shift in the position of the OPWs along the gut tract. From these observations, the authors suggested that the enteric nervous system may be involved in the motility at earlier stages than previously reported (E16) (Chevalier et al., 2019). Here, I show by simulation that OPWs can emerge spontaneously in smooth muscle tissue that has a high probability of spontaneous depolarization and an associated refractory period, without need for pacemaker or neurogenic activity. The simulations additionally demonstrates that pacemaking by Interstitial Cells of Cajal requires a developmental decrease of the spontaneous depolarization probability of the smooth muscle syncytium.

Localized above-threshold depolarization of the smooth muscle leads to the nucleation of two contractile waves propagating symmetrically away from the point of depolarization, at a constant speed of about 30 μm/s at E8-E12 (Chevalier et al., 2020; Shikaya et al., 2022), and that further depolarizes the tissue in its wake. Smooth muscle can only spontaneously depolarize again after a certain refractory period, which is typically on the order of 10–60 s for chicken embryonic intestinal smooth muscle. The simple idea behind the simulations I present here is that the most probable site for the nucleation of a second wave is the site of the first nucleation, because it is the point where most time has elapsed since it last depolarized. To implement this idea, I put together a Matlab program for a discretized gut (*N* = 200 sites), with the following rules:

• At each time-step, each site can self-depolarize with a probability p

• If self-depolarized, a site gives rise to 2 contractile (depolarizing) waves propagating symmetrically away from the point of depolarization, at a constant speed of 30 μm/s.
• A site can only depolarize after a refractory time τ has elapsed since it last contracted.
• Colliding waves annihilate, as is observed experimentally.

**FIGURE 1:**
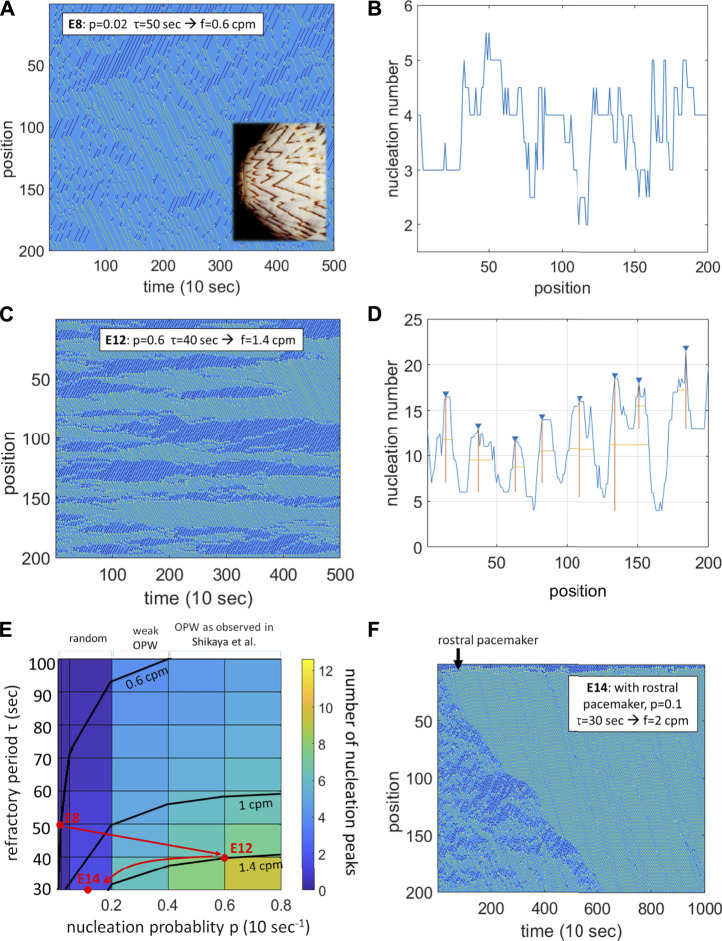
A probabilistic refractory period model of the intestinal smooth muscle syncitium reproduces the contractile wave patterns observed in chicken at E8, E12 and E14. **(A)** Spatiotemporal map for *p* = 0.02, τ = 50 s, yielding *f* = 0.6 cpm. Rostro-caudal waves in green, caudo-rostral waves in dark blue, nucleation sites in yellow. Inset: the wave pattern is reminiscent of the pigmentation of some seashells (Meinhardt and Klingler, 1987). **(B)** Homogeneous distribution of nucleation sites along the gut corresponding to **(A)**. **(C)** Spatiotemporal map for *p* = 0.4, *τ* = 40 s, yielding *f* = 1.4 cpm. **(D)** Peaked distribution of nucleation sites (OPWs) along the gut corresponding to **(C)**. **(E)** Color-coded map of the average number of nucleation sites (OPWs) as a function of the refractory period *τ* and of the nucleation probability *p*. Frequency isolines at 0.6, 1 and 1.4 cpm are shown. Red dots: parameters used in **(A,C,F)** with the suggested developmental trajectory. **(F)** Spatiotemporal map for *p* = 0.1, *τ* = 30 s with an additional rostral pacemaker $p_{rostral}=cos\left(2\pi ft\right)$ with *f* = 2 cpm. The diagram is progressively invaded by rostro-caudal waves from the pacemaker site when the nucleation probability in the rest of the gut smooth muscle is low enough.

The simulation was run over 500 time steps, and parameters p and τ were explored to reproduce the experimentally observed frequencies of contractiles waves f of 0.6 cpm (cycles per minute) at E8 (Chevalier et al., 2017; Shikaya et al., 2022) and of 1.4 cpm at E12 (Chevalier et al., 2020; Shikaya et al., 2022). At E8 ( f=0.6 cpm), I observed random distribution of nucleation sites along the length of the gut for all values of *τ* < 60 s (Figures 1A,B), in agreement with the findings of Shikaya et al. (2022) at this stage. The wave pattern is reminiscent of that of the pigmentation of some seashells (inset), that occurs by a similar traveling wave mechanism (Meinhardt and Klingler, 1987). At E12 ( f=1.4 cpm), nucleation sites concentrated along distinct positions along the length of the gut (Figures 1C,D). The spatiotemporal maps and the spontaneous emergence of OPWs strongly resemble the results of Shikaya et al. (2022) The positions of the OPWs slowly drifted with time and could even disappear (Figure 1C). A similar continuous drift occurring both before and after the administration of tetrodotoxin was reported in Figure 5B by Shikaya et al. (2022)—because the drift is continuous I do not think it is induced by tetrodotoxin but rather reflects the natural time evolution of a purely myogenic system. Concerning the observed shift of OPWs in ENS-ablated guts, I note that it is only observed in the first half of the midgut, and that it only concerns 2 peaks out of the 4 in this region; Supplementary Figure S3 of Shikaya et al. (2022) shows that such OPWs located outside of the 8 regions they defined can occur frequently in control guts as well. Figure 1E summarizes the regions where OPWs are observed (color coded) as a function of p and τ , and the constant frequency lines at f=0.6 cpm and f = 1.4 cpm.

The spatiotemporal diagram changes drastically at E14 as pacemaker interstitial cells of Cajal start being active (Chevalier et al., 2020), with waves becoming more unidirectional and regular in time. I modeled this by implementing an oscillatory probability of nucleation at the rostral end $p_{rostral}=\text{cos}\left(2\pi ft\right)$ with *f* = 2 cpm at E14 (Chevalier et al., 2020), and *τ* = 30 s (to accommodate the pacemaker frequency). Interestingly, I found that the spontaneous depolarization probability of the muscle had to decrease from its value at E12 (from ∼0.4 to ∼0.1) in order for the rostral pacemaker to invade the spatiotemporal diagram (Figure 1F), as is experimentally observed (Chevalier et al., 2020). This developmental trajectory is summarized in Figure 1E. This result may indicate that the smooth muscle cells have to loose their self-depolarizing properties in order for pacemaking by ICCs to ensue.
